# Evaluation of Socioeconomic Position and Survival After Out-of-Hospital Cardiac Arrest in Korea Using Structural Equation Modeling

**DOI:** 10.1001/jamanetworkopen.2023.12722

**Published:** 2023-05-10

**Authors:** Dong Hyun Choi, Young Sun Ro, Jeong Ho Park, Sun Young Lee, Ki Jeong Hong, Kyoung Jun Song, Sang Do Shin

**Affiliations:** 1 Laboratory of Emergency Medical Services, Seoul National University Hospital Biomedical Research Institute, Seoul, Korea; 2 Department of Biomedical Engineering, Seoul National University College of Medicine, Seoul, Korea; 3 Department of Emergency Medicine, Seoul National University Hospital, Seoul, Korea; 4 Department of Emergency Medicine, Seoul National University College of Medicine, Seoul, Korea; 5 Public Healthcare Center, Seoul National University Hospital, Seoul, Korea; 6 Department of Emergency Medicine, Seoul National University Boramae Medical Center, Seoul, Korea

## Abstract

**Importance:**

The association between low socioeconomic position (SEP) and poor survival after out-of-hospital cardiac arrest (OHCA) has not been thoroughly investigated.

**Objectives:**

To evaluate the association between individual SEP and survival after OHCA and to identify any mediating pathways using structural equation modeling (SEM).

**Design, Setting, and Participants:**

This is a retrospective cohort study that used data collected from January 2013 to December 2019. Participants were adults with OHCA with a presumed cardiac etiology. The study was conducted in Korea, which has a universal health insurance system. Data were analyzed from January 2022 to February 2023.

**Exposures:**

Individual SEP was measured by insurance type (National Health Insurance [NHI] and medical aid [MA]) and premiums. SEP was categorized into 5 groups, in which NHI beneficiaries were divided into quartiles (Q1, highest quartile; Q4, lowest quartile), and MA was the lowest SEP group.

**Main Outcomes and Measures:**

The primary outcome was survival to discharge. The association between SEP and OHCA survival was examined using multivariable logistic regression, and mediating factors were identified using SEM.

**Results:**

A total of 121 516 patients (median [IQR] age, 73 [60-81] years; 43 912 [36.1%] female patients) were included. Compared with the NHI Q1 group, individuals with lower SEP had lower odds of survival to discharge. The adjusted odds ratios of survival to discharge were 0.97 (95% CI, 0.94-1.00), 0.88 (95% CI, 0.85-0.91), 0.91 (95% CI, 0.88-0.94), and 0.53 (95% CI, 0.50-0.56) for the NHI Q2, NHI Q3, NHI Q4, and MA groups, respectively. Several factors were found to mediate the association of SEP and survival in the total study population, with mediating proportions of 15.1% (95% CI, 11.8%-18.4%) for witnessed status, 4.8% (95% CI, 3.5%-6.0%) for bystander cardiopulmonary resuscitation provision, 41.8% (95% CI, 35.4%-48.1%) for initial rhythm, and 9.4% (95% CI, 7.4%-11.4%) for emergency department level. Among patients who survived to hospital admission, the mediation proportions were 11.8% (95% CI, 6.7%-16.9%) for witnessed status, 3.7% (95% CI, 1.3%-6.1%) for bystander cardiopulmonary resuscitation provision, 56.2% (95% CI, 41.0%-71.4%) for initial rhythm, 10.7% (95% CI, 6.1%-15.3%) for emergency department level, 20.2% (95% CI, 14.0%-26.5%) for coronary angiography, and 4.2% (95% CI, 2.2%-6.1%) for targeted temperature management.

**Conclusions and Relevance:**

In this cohort study of patients with OHCA, lower individual SEP was significantly associated with lower survival to discharge. Potentially modifiable mediators can be targeted for public health interventions to reduce disparities in survival among patients with OHCA of different SEP.

## Key Points

**Question:**

What is the association between socioeconomic position (SEP) and survival after out-of-hospital cardiac arrest (OHCA), and what are possible underlying mechanisms?

**Findings:**

In this cohort study of 121 516 Korean adults with OHCA, lower SEP was associated with lower odds of survival to discharge. The mediation proportions for witnessed status, bystander cardiopulmonary resuscitation, and emergency department level in all patients were 15.1%, 4.8%, and 9.4%, respectively, while those for coronary angiography and targeted temperature management in admitted patients were 20.2% and 4.2%, respectively.

**Meaning:**

These findings suggest that public health interventions targeting potentially modifiable mediators can be implemented to reduce disparities in survival among patients with OHCA with different SEP.

## Teaser

This cohort study evaluates the association and mediating pathways between individual socioeconomic position and survival after out-of-hospital cardiac arrest (OHCA) among adults in Korea.

## Introduction

The incidence of emergency medical services (EMS)–treated out-of-hospital cardiac arrest (OHCA) is nearly 34.7 per 100 000 person-years and is a leading cause of death globally.^1^ The survival to discharge rate of patients with OHCA has significantly increased during the previous decades but generally remains less than 10%.^2^ In addition, there are significant variations in the survival to discharge rate between different communities, countries, and continents (range, 4.5%-16.2%).^3^ Survival after OHCA is influenced by multiple factors, including patient characteristics, circumstances of the OHCA event, bystander and EMS interventions, and in-hospital care.^4^ Among these factors, those related to bystanders, EMS, and in-hospital care on the chain of survival are considered potentially modifiable through public health policies and intervention programs.^5^

Recently, there have been many reports that socioeconomic position (SEP), measured by income, education level, or employment status, is associated with the incidence of OHCA and survival of patients with OHCA.^6^ Most studies observed that a low SEP was associated with a higher OHCA incidence and lower rates of survival.^6,7^ Differences in patient characteristics and disparities in each component of the chain of survival are assumed to contribute to the association between SEP and survival after OHCA. Patients with OHCA in a low-income neighborhood were less likely to receive bystander cardiopulmonary resuscitation (CPR) than those in high-income neighborhoods.^8^ Lower area-level SEP, measured by property tax per capita of the region, was associated with a longer EMS response time in patients with OHCA.^9^ In addition, patients with low individual income were less likely to receive coronary angiography (CAG) after successful resuscitation from OHCA.^10^

Mediation analysis aims to identify the mechanism between the independent and dependent variables through mediator variables. Thus, associations without an obvious direct connection can be better understood, and mediating variables can be targeted for interventions.^11^ However, the mediating pathways between SEP and survival after OHCA have not been thoroughly investigated.

Identifying the mechanism between SEP and survival after OHCA can contribute to the development of targeted interventions and health policies to decrease disparities in survival outcomes. In addition, disentangling pathways and calculating the proportion of the variation explained by each mediator can aid in deciding priorities in the interventions. This study has 2 aims. First, we aimed to evaluate the association between individual SEP and survival to hospital discharge in patients with OHCA using a nationwide universal health insurance system database. Second, we aimed to identify the mediators and pathways of this association and measure the extent of the variation explained through each mediator using structural equation modeling (SEM).

## Methods

### Study Design and Setting

This retrospective cohort study used a nationwide OHCA registry linked to the National Health Insurance Database (NHID) in Korea. This study was approved by the institutional review boards of Seoul National University Hospital, and the requirement for informed consent was waived due to the study’s retrospective design and because patient information was anonymized before the analysis. The National Health Insurance Service also approved this study. We followed the Strengthening the Reporting of Observational Studies in Epidemiology (STROBE) guidelines.

The universal health insurance system in Korea comprises 2 types of insurance: National Health Insurance (NHI) and medical aid (MA). NHI covers most Korean nationals who reside within the country (97.0% in 2015), excluding those supported by MA (3.0%).^12^ NHI premiums are levied based on the household income (including property and other assets) of the beneficiaries.^13^ The beneficiaries of MA are individuals with very low SEP, including those whose household income is less than 40% of the standard median income necessary for 1 person to live a healthy and decent life (approximately $600 per month for a single-person household in 2015).^14^ The MA system is run by tax, and beneficiaries are not charged any premium. Since insurance is based on income, insurance type and premiums have been widely used as a proxy for individual SEP.^13^

Patients who receive a certain hospital treatment covered by insurance pay a portion of the costs as copayments. While the copayment rate differs by level of care, it is generally 5% to 20% for patients with NHI and 0% to 5% for patient with MA admitted to the hospital.^14^ The coverage ranges of treatments provided by NHI and MA services are almost similar. While most cardiac arrest–related care is covered by both insurances, certain stenting devices and sealing materials for percutaneous coronary interventions may not be fully covered by either insurance.^14^ Additionally, until July 2019, neither insurance covered the mechanical surface cooling device used for targeted temperature management (TTM).

The Korean EMS is a single-tiered government-based system operated by 16 provincial headquarters in the national fire department. The Korean EMS is tax-financed and free of charge for all patients. It covers an entire Korean population of approximately 50 million people across 100 210 km^2^. EMS personnel can provide basic to intermediate levels of care for patients with OHCA, including CPR, advanced airway management, intravenous fluids, and epinephrine injections under direct medical oversight. Since EMS personnel cannot declare death in the field unless there are signs of irreversible death, all patients with OHCA are transported to the nearest emergency department (ED). EDs are designated as levels 1 to 3: level 1 (38 facilities) and level 2 (119 facilities) have the highest volumes, with emergency physicians staffed at all times, and level 3 (261 facilities) can be staffed by general physicians.

### Data Sources

The NHID contains data on the entire Korean population for both NHI and MA beneficiaries. Demographic characteristics, insurance-related data, and all inpatient and outpatient diagnoses are included in the NHID. The NHID was linked with the Korean OHCA registry using the individual’s unique resident registration number.

The Korean OHCA registry was first developed in 2006 and includes all patients in Korea with OHCA who were treated by EMS. Data from EMS run sheets, EMS OHCA registry, and hospital medical record review were merged into a single registry. EMS run sheets and EMS OHCA registry, which were recorded by EMS professionals, contain data on patient demographic characteristics, circumstances of the OHCA event, and EMS treatment. In-hospital care and clinical outcome data of patients with OHCA were extracted by 6 to 8 trained Korean Centers for Disease Control and Prevention medical record reviewers. Quality control of the Korean OHCA registry is performed monthly, with feedback provided to the medical record reviewers.^15^

### Study Population

All adult (aged ≥19 years) patients with OHCA of presumed cardiac etiology from January 2013 to December 2019 were included in the analysis. Patients with noncardiac etiology, including asphyxia, drowning, trauma, poisoning, or burns, were excluded from this study. Those who were not matched to the NHID or had missing insurance type or premium data were also excluded.

### Study Outcomes

The primary outcome of this study was survival to discharge. The secondary outcome was good neurological recovery, defined as a cerebral performance category score of 1 (good cerebral performance) or 2 (moderate cerebral disability) at the time of hospital discharge.

### Variables and Measurements

The main exposure of this study was the SEP level categorized into 5 groups (NHI Q1, NHI Q2, NHI Q3, NHI Q4, and MA) according to the type of insurance and NHI premiums 1 year prior to the index date. NHI Q1 indicates beneficiaries with NHI premiums in the highest quartile, and NHI Q4 indicates beneficiaries with NHI premiums in the lowest quartile among the total Korean NHI beneficiary population.^16^ NHI Q1 was the highest SEP group, while MA was the lowest SEP group, similar to a recently published study.^17^ In the year 2015, the mean annual household incomes for the NHI Q1, NHI Q2, NHI Q3, and NHI Q4 groups, which include property and other assets, were approximately $62 000, $41 000, $29 500, and $27 000, respectively.^18^ Type of insurance, NHI premiums, age, sex, residential region, and comorbidities were extracted from the NHID. Location of arrest, witnessed status, bystander CPR, bystander automated external defibrillator (AED) use, response time interval (RTI), initial rhythm, receiving ED level, CAG, TTM, and hospital outcomes were obtained from the Korean OHCA registry.

### Statistical Analysis

Descriptive analysis was conducted to compare the characteristics of the study population. Imputation of missing data on the variable location of arrest (missing proportion, 2.9%), witnessed status (9.0%), bystander CPR (2.5%), bystander AED use (1.4%), RTI (0.2%), and initial rhythm (0.6%) was performed by stochastic regression imputation using logistic regression (eTable 1 in Supplement 1).^19^ *P* values were based on a 2-sided significance level of .05.

Multivariable logistic regression analysis was performed to examine the association between SEP and hospital outcomes in patients with OHCA. Crude and adjusted odds ratios (ORs) with 95% CIs were calculated adjusting for potential confounders. Based on the literature review, variables that are determined prior to the exposure (SEP) and can potentially affect both the exposure (SEP) and the outcome (survival to discharge) were selected as potential confounders.^20,21^

Mediation analysis and SEM were performed for the binarized SEP groups using various thresholds: MA vs NHI Q1 to Q4; MA and NHI Q4 vs NHI Q1 to Q3; MA, NHI Q3, and NIH Q4 vs NHI Q1 and NHI Q2; and MA and NHI Q2 to Q4 vs NHI Q1. The main results were presented based on binarizing SEP groups into MA vs NHI Q1 to Q4, as this threshold showed the most distinguished odds of survival to discharge. To investigate whether a variable is a mediator of the SEP-OHCA survival association according to the method of Baron and Kenny, multivariable logistic regression was used.^22^ Based on the literature review, variables that were determined after the exposure (SEP) and could potentially affect the outcome (survival to discharge) were selected as mediators.

SEM is a general statistical framework for simultaneous model regression equations and tests the hypothetical associations between many variables.^23^ SEM was fitted using the robust maximum likelihood estimation method, which is known to perform well in SEM with categorical variables.^24^ Age, sex, diabetes, hypertension, and residential region were included as confounders in the SEM. A hypothetical model from SEP to OHCA survival to discharge was constructed based on prior knowledge and temporal order (eMethods in Supplement 1). The goodness of fit was assessed using root mean square error of approximation (RMSEA), standardized root mean square residual (SRMR), goodness of fit index (GFI), and comparative fit index (CFI).^25^ The mediation proportions through specific pathways were analyzed using the product of coefficients approach.^26^ The mediation proportion captures how important a specific pathway through a mediator is in explaining the total association.^26,27^

A sensitivity analysis was performed on patients with OHCA who survived to hospital admission to measure the proportion of mediation for in-hospital treatments, such as CAG and TTM. All statistical analyses were conducted using SAS version 9.4 (SAS Institute Inc).

## Results

Among the 196 422 patients with OHCA treated by EMS during the study period, 121 516 (median [IQR] age, 73 [60–81] years; 43 912 [36.1%] female patients) were eligible for the analysis (eFigure 1 in Supplement 1). There were 40 483, 26 955, 21 625, 22 201, and 10 252 patients in the NHI Q1, NHI Q2, NHI Q3, NHI Q4, and MA groups, respectively. Notable differences in witnessed arrest (MA, 4378 [42.7%]; NHI Q1, 20 090 [49.6%]), bystander CPR provision (MA, 5826 [56.8%]; NHI Q1, 24 971 [61.7%]), initial shockable rhythm (MA, 990 [9.7%]; NHI Q1, 5690 [14.1%]), and ED level 1 or 2 (MA, 5939 [57.9%]; NHI Q1, 26 384 [65.2%]) were observed between the SEP groups. The proportion of patients who survived to discharge and had good neurological recovery were 4.5% (465 patients) and 2.1% (216 patients) in the MA group, respectively, and 7.2% (2916 patients) and 4.6% (1842 patients) in the NHI Q1 group, respectively (Table 1).

**Table 1.** Characteristics and Outcomes of the Total Study Population According to Individual Socioeconomic Position

Characteristic	Patients by socioeconomic position, No. (%)					*P* value
	NHI				MA (n = 10 252)	
	Q1 (n = 40 483)	Q2 (n = 26 955)	Q3 (n = 21 625)	Q4 (n = 22 201)		
Age, median (IQR), y	77 (66-83)	71 (59-80)	67 (55-78)	70 (58-79)	74 (59-82)	<.001
Standardized mean difference	[Reference]	−0.33	−0.52	−0.37	−0.21	NA
Aged 65-120 y	31 254 (77.2)	17 595 (65.3)	11 895 (55.0)	13 638 (61.4)	6734 (65.7)	<.001
Sex						
Female	15 006 (37.1)	8988 (33.3)	6982 (32.3)	7866 (35.4)	5070 (49.5)	<.001
Male	25 477 (62.9)	17 967 (66.7)	14 643 (67.7)	14 335 (64.6)	5182 (50.5)	
Diabetes	10 346 (25.6)	6451 (23.9)	5046 (23.3)	5301 (23.9)	2756 (26.9)	<.001
Hypertension	21 185 (52.3)	12 898 (47.9)	9781 (45.2)	10 625 (47.9)	5307 (51.8)	<.001
Residential region, metropolitan	17 594 (43.5)	11 385 (42.2)	9254 (42.8)	9525 (42.9)	4207 (41.0)	<.001
Location of arrest, public	5734 (14.2)	4221 (15.7)	3781 (17.5)	3915 (17.6)	1087 (10.6)	<.001
Witnessed arrest	20 090 (49.6)	13 225 (49.1)	10 392 (48.1)	10 717 (48.3)	4378 (42.7)	<.001
Bystander CPR	24 971 (61.7)	16 487 (61.2)	13 161 (60.9)	13 397 (60.3)	5826 (56.8)	<.001
Bystander AED use	1014 (2.5)	654 (2.4)	529 (2.4)	512 (2.3)	250 (2.4)	.47
RTI, median (IQR), min	7 (5-10)	7 (5-10)	7 (5-10)	7 (5-10)	7 (5-10)	.002
Standardized mean difference	[Reference]	0.02	0.01	−0.01	−0.01	NA
RTI <8 min	23 158 (57.2)	15 247 (56.6)	12 217 (56.5)	12 867 (58.0)	5917 (57.7)	.009
Initial shockable rhythm	5690 (14.1)	4561 (16.9)	3771 (17.4)	3723 (16.8)	990 (9.7)	<.001
ED level 1-2	26 384 (65.2)	17 074 (63.3)	13 765 (63.7)	14 339 (64.6)	5939 (57.9)	<.001
CAG	1872 (4.6)	1432 (5.3)	1211 (5.6)	1172 (5.3)	232 (2.3)	<.001
TTM	1173 (2.9)	817 (3.0)	721 (3.3)	670 (3.0)	161 (1.6)	<.001
Survival to admission	7680 (19.0)	5546 (20.6)	4683 (21.7)	4711 (21.2)	1625 (15.9)	<.001
Survival to discharge	2916 (7.2)	2250 (8.3)	1880 (8.7)	1832 (8.3)	465 (4.5)	<.001
Good neurological recovery	1842 (4.6)	1458 (5.4)	1250 (5.8)	1108 (5.0)	216 (2.1)	<.001

Abbreviations: AED, automated external defibrillator; CAG, coronary angiography; CPR, cardiopulmonary resuscitation; ED, emergency department; MA, medical aid; NA, not applicable; NHI, National Health Insurance; Q, quartile; RTI, response time interval; TTM, targeted temperature management.

**Figure 1.**
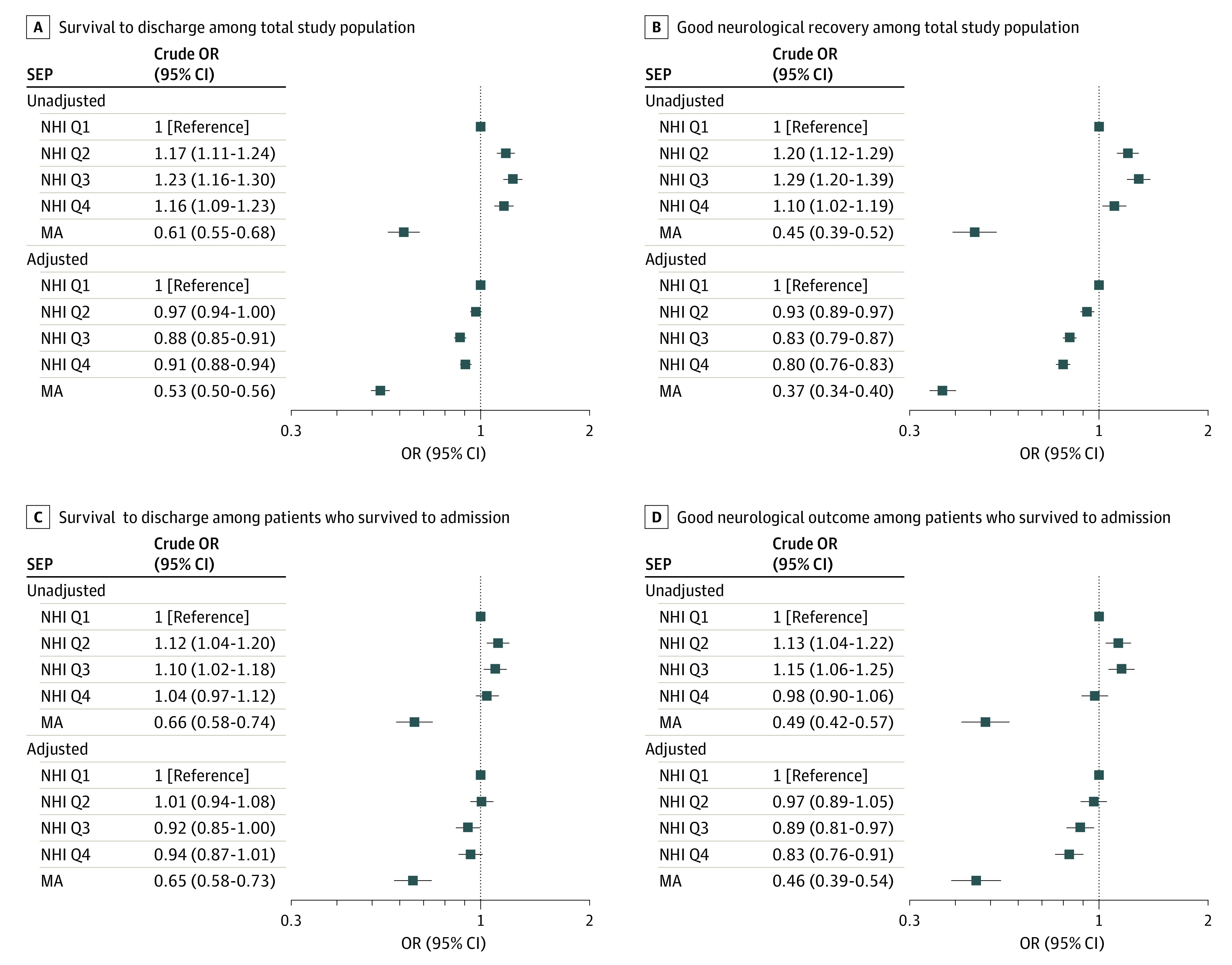
Association Between Individual Socioeconomic Position and Hospital Outcomes Among Patients With Out-of-Hospital Cardiac Arrest Adjusted odds ratios (ORs) were calculated with a multivariable logistic regression model adjusting for age, sex, hypertension, diabetes mellitus, and residential region. NHI indicates National Health Insurance; MA, medical aid.

Compared with that of the NHI Q1 group, the adjusted ORs of survival to discharge and good neurological recovery were shown in Figure 1. Compared with the NHI Q1 group, the adjusted odds ratio of survival to discharge was 0.97 (95% CI, 0.94-1.00), 0.88 (95% CI, 0.85-0.91), 0.91 (95% CI, 0.88-0.94), and 0.53 (95% CI, 0.50-0.56) for the NHI Q2, NHI Q3, NHI Q4, and MA groups, respectively. The MA group was significantly less likely to survive to discharge (adjusted OR, 0.56 [95% CI, 0.53-0.59]) and achieve good neurological recovery (adjusted OR, 0.41 [95% CI, 0.38-0.45]) compared with that of the NHI Q1 to Q4 groups (eTable 2 in Supplement 1).

**Figure 2.**
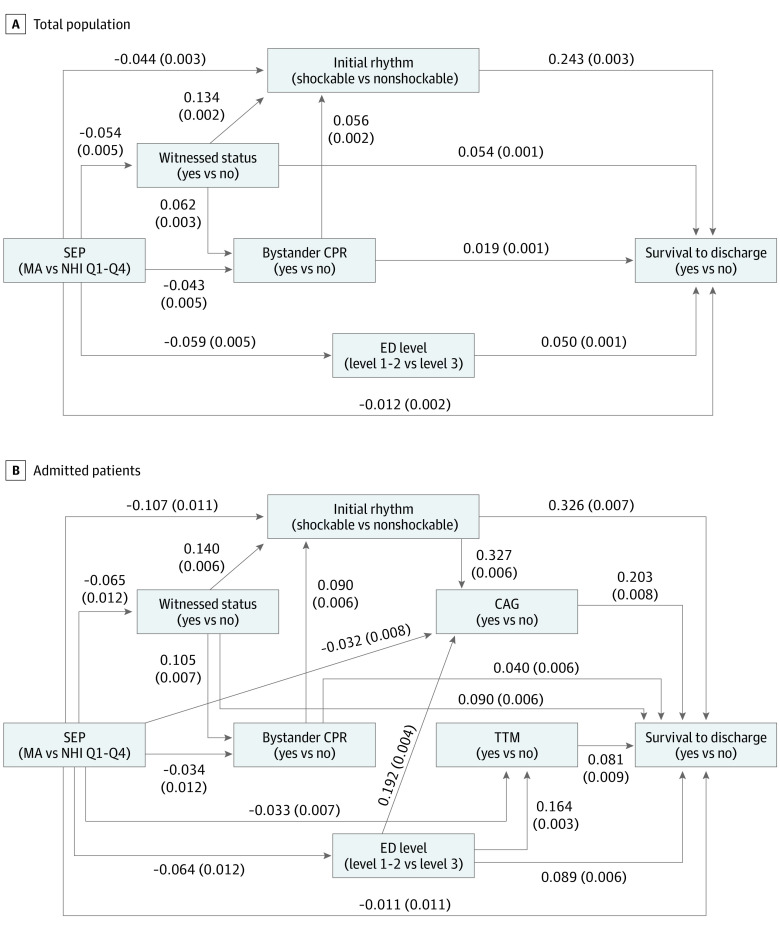
Structural Equation Modeling Diagram from Socioeconomic Position (SEP) to Survival to Discharge Coefficients (SEs) of pathways between SEP, survival to discharge, and mediators are shown in the figure. CAG indicates coronary angiography; CPR, cardiopulmonary resuscitation; ED, emergency department; MA, medical aid; NHI, National Health Insurance; Q, quartile; TTM, targeted temperature management.

Witnessed status, bystander CPR, initial rhythm, and ED level were identified as mediators of the SEP and survival to discharge after OHCA association in the total study population (eTable 3 in Supplement 1). The SEM path analysis results (MA group vs NHI Q1-Q4 groups) are shown in Figure 2. Goodness of fit indices (RMSEA, 0.056; SRMR, 0.013; GFI, 1.00; and CFI, 0.98) indicated that the model was well fitted.^25^ The mediation proportion was 15.1% (95% CI, 11.8%-18.4%) for witnessed status, 4.8% (95% CI, 3.5%-6.0%) for bystander CPR, 41.8% (95% CI, 35.4%-48.1%) for initial rhythm, and 9.4% (95% CI, 7.4%-11.4%) for ED level. The proportions mediated through bystander CPR and initial rhythm without passing through its intermediate confounders in the pathway of SEP-mediator-OHCA survival (ie, the variables affected by the exposure variable, which in turn affects both of the mediator and the outcome variable) were 4.4% (95% CI, 3.2%-5.6%) and 34.2% (95% CI, 28.4%-40.0%), respectively (Table 2). These proportions could be interpreted as percentage changes in the total size of the association between SEP on OHCA survival if we could disable the direct pathways from the SEP to the mediators. Results were generally consistent across different SEP binarization cutoffs in which the mediation proportions ranged as follows: 15.1% to 23.5% for witnessed status, 4.8% to 9.0% for bystander CPR, up to 41.8% for initial shockable rhythm, and 7.9% to 16.6% for ED level (eTable 4 and eFigure 2 in Supplement 1).

**Table 2.** Estimates of Pathways in the Association Between Socioeconomic Position (Medical Aid vs National Health Insurance Quartiles 1-4) and Survival to Discharge Among the Total Study Population

Pathway	Effect estimate (95% CI)	Mediation proportion (95% CI), %
Total	−0.032 (−0.036 to −0.027)	NA
Witnessed status	−0.005 (−0.006 to −0.004)	15.1 (11.8 to 18.4)
Bystander CPR	−0.002 (−0.002 to −0.001)	4.8 (3.5 to 6.0)
Bystander CPR but not through witnessed status^a^	−0.001 (−0.002 to −0.001)	4.4 (3.2 to 5.6)
Initial rhythm	−0.013 (−0.015 to −0.012)	41.8 (35.4 to 48.1)
Initial rhythm but not through witnessed status or bystander CPR^a^	−0.011 (−0.012 to −0.009)	34.2 (28.4 to 40.0)
ED level	−0.003 (−0.003 to −0.002)	9.4 (7.4 to 11.4)
Direct	−0.012 (−0.016 to −0.008)	36.9 (28.1 to 45.7)

Abbreviations: CPR, cardiopulmonary resuscitation; ED, emergency department; NA, not applicable

^a^ The proportion mediated through a mediator but not through its intermediate confounders (variable that is affected by the exposure, which in turn affects the outcome and mediator) is assessed.

In the sensitivity analysis of patients who survived to hospital admission (eTable 5 in Supplement 1), witnessed status, bystander CPR, initial rhythm, ED level, CAG, and TTM were identified as mediators (eTable 6 in Supplement 1). The SEM of MA group vs NHI Q1 to Q4 groups as shown in Figure 2 was well fitted (RMSEA, 0.048; SRMR, 0.019; GFI, 1.00; and CFI = 0.98). The mediation proportion was 11.8% (95% CI, 6.7%-16.9%) for witnessed status, 3.7% (95% CI, 1.3%-6.1%) for bystander CPR, 56.2% (95% CI, 41.0%-71.4%) for initial rhythm, 10.7% (95% CI, 6.1%-15.3%) for ED level, 20.2% (95% CI, 14.0%-26.5%) for CAG, and 4.2% (95% CI, 2.2%-6.1%) for TTM (Table 3). The mediation proportions across different SEP binarization cutoffs are presented in eTable 7 and eFigure 3 in Supplement 1.

**Table 3.** Estimates of Pathways in the Association Between Socioeconomic Position (Medical Aid vs National Health Insurance Quartiles 1-4) and Survival to Discharge Among Patients Who Survived to Hospital Admission

Pathway	Effect estimate (95% CI)	Mediation proportion (95% CI), %
Total	−0.084 (−0.107 to −0.061)	NA
Witnessed status	−0.010 (−0.014 to −0.006)	11.8 (6.7 to 16.9)
Bystander CPR	−0.003 (−0.005 to −0.001)	3.7 (1.3 to 6.1)
Bystander CPR but not through witnessed status^a^	−0.003 (−0.004 to −0.001)	3.1 (0.8 to 5.4)
Initial rhythm	−0.047 (−0.056 to −0.039)	56.2 (41.0 to 71.4)
Initial rhythm but not through witnessed status or bystander CPR^a^	−0.042 (−0.051 to −0.034)	50.2 (36.1 to 64.3)
ED level	−0.009 (−0.012 to −0.006)	10.7 (6.1 to 15.3)
CAG	−0.017 (−0.021 to −0.013)	20.2 (14.0 to 26.5)
CAG but not through initial rhythm or ED level^a^	−0.007 (−0.010 to −0.003)	7.8 (3.4 to 12.2)
TTM	−0.004 (−0.005 to −0.002)	4.2 (2.2 to 6.1)
TTM but not through ED level^a^	−0.003 (−0.004 to −0.001)	3.2 (1.4 to 4.9)
Direct	−0.011 (−0.032 to 0.010)	NA^b^

Abbreviations: CAG, coronary angiography; CPR, cardiopulmonary resuscitation; ED, emergency department; NA, not applicable; TTM, targeted temperature management.

^a^ The proportion mediated through a mediator but not through its intermediate confounders (variable that is affected by the exposure, which in turn affects the outcome and mediator) is assessed.

^b^ The mediation proportions are not calculated for nonsignificant estimates.

## Discussion

This study, which used a nationwide universal health insurance system database, found that lower individual SEP was associated with lower survival to discharge after OHCA (adjusted OR, 0.53 [95% CI, 0.50-0.56]) for the MA group, compared with the NHI Q1 group. Using SEM, the pathways from SEP to OHCA survival were modeled with identified mediators. The mediation proportions were measured in the total study population (witnessed status, 15.1%; bystander CPR, 4.8%; initial rhythm, 41.8%; ED level, 9.4%) and in patients who survived to admission (witnessed status, 11.8%; bystander CPR, 3.7%; initial rhythm, 56.2%; ED level, 10.7%; CAG, 20.2%; TTM, 4.2%). The SEM analysis showed significant direct and indirect pathways from SEP to OHCA survival, indicating that these indirect pathways with potentially modifiable mediators could be the targets of public health interventions.

This study used nationally representative data from Korea, which has a universal health insurance system, and this study is one of the largest studies for patients with OHCA (N = 121 516) to date to explore the association between SEP and OHCA survival outcomes. To our knowledge, this is the first study to use SEM to model and disentangle the pathways from the SEP to survival after OHCA with multiple mediators.

Previous literature reported differences in the provision of bystander CPR according to SEP.^7^ The mediation proportion of bystander CPR in this study was not negligible (4.8%), but a recent Danish study found that the proportion of the SEP-OHCA survival association explained by bystander CPR was less than 1%.^21^ Efforts to increase public OHCA awareness and CPR training are required. The proportion mediated through witnessed status in this study was quite high (15.1%), although witnessed status has not received much attention as a mediator previously. A solitary lifestyle and poor awareness of prodromal symptoms may be one of the explanations,^28,29^ although these factors were not measured in this study. Whether witness status can be regarded as a modifiable risk factor is debatable. A campaign in Australia to increase public awareness of the warning signs of cardiac arrest reduced the proportion of unwitnessed OHCA while the proportion of witnessed OHCA did not change.^30^ Witnessed status may be modifiable through public education on the recognition of prodromal symptoms and emphasizing the importance of early EMS activation.^31^

Among patients who survived to hospital admission, a significant portion of the SEP disparities was explained by the pathways through CAG and TTM without passing through other mediators. This suggests that patients with different SEP receive varying levels of post–cardiac arrest care, even if the proportion of patients with an initial shockable rhythm and the proportion of patients treated at high-level EDs are similar. Some of the coronary stenting devices and mechanical cooling devices for TTM are not covered by insurance, which may cause hesitation among conscious patients, guardians, and physicians to start costly treatments that are not covered. Therefore, public health interventions aimed at increasing the insurance coverage of MA beneficiaries should be considered to reduce this gap.

The initial rhythm on the electrocardiogram was found to be an important mediator of the SEP-OHCA survival association. Previous studies have suggested that favorable resuscitation characteristics, including witnessed arrest and shorter response time, are associated with initial shockable rhythm.^20,32^ Differences in the etiology of cardiogenic cardiac arrest and disparities in the no-flow time to recognition of OHCA may have also contributed to the association between SEP and initial rhythm. Although the initial rhythm is not directly modifiable, interventions to reduce disparities in witnessed status, bystander CPR provision, and no-flow time may be effective. Bystander AED use and RTI were not significant mediators of the SEP-OHCA survival association in this study. However, the outcomes of these mediators may be changed by the prevalence of bystander AED use and EMS ambulance dispatch protocols.

### Limitations

This study has some limitations that should be considered when interpreting the results. First, this study is limited by its retrospective nature with potential of unmeasured biases. Second, there may be unmeasured confounders and mediators of the SEP-OHCA survival association. Third, insurance premiums may not perfectly reflect a patient’s individual SEP. In addition, the insurance premium 1 year before the index date was used, which may differ from the lifelong SEP of patients with OHCA. Fourth, we did not consider exposure-mediator and mediator-mediator interactions. This limitation also applies to previous studies that assessed the association between SEP and survival after OHCA.^20,21^ Fifth, the significant and well-fitted results of the SEM analysis do not verify that the associations in the hypothetical model are causal. Sixth, approximately 22 000 patients who were not matched or had missing insurance information were excluded, and most of them lacked a resident registration number in the database. It is possible that the lack of registration numbers did not occur randomly, which could lead to bias in the results. Seventh, although we performed imputation assuming that data were missing at random, there may have been unknown factors associated with the missingness that were not accounted for. Finally, the results of this study may not be generalizable to other settings with different EMS and insurance systems. Differences in the prevalence of ST-elevation myocardial infarction and differences in local practices of performing CAG and TTM may also limit the generalizability of this study.

## Conclusions

In this study, lower individual SEP was significantly associated with lower survival to discharge after OHCA. Pathways through potentially modifiable mediators, including witnessed status, bystander CPR, ED level, and postresuscitation care (ie, CAG and TTM), can be targets for public health interventions to reduce disparities in survival outcomes among patients with OHCA of different SEP.

## Notes

Supplement 1.
eMethods.

eReferences.
**eTable 1.** Percentage of Missing Data for Each Variable **eTable 2.** Association Between Individual SEP and Hospital Outcomes in the Total Study Population **eTable 3.** Mediation Analysis on the Association Between SEP and Survival to Discharge After OHCA (Total Study Population) **eTable 4.** Estimates of Pathways in the SEP and Survival to Discharge Association (Total Study Population) **eTable 5.** Characteristics and Outcomes of Patients Who Survived to Admission According to Individual SEP **eTable 6.** Mediation Analysis on the Association Between SEP and Survival to Discharge After OHCA (Patients Who Survived to Admission) **eTable 7.** Estimates of Pathways in the SEP and Survival to Discharge Association (Patients Who Survived to Admission) **eFigure 1.** Study Flowchart **eFigure 2.** Structural Equation Modeling Diagram From SEP to Survival to Discharge in Total Study Population **eFigure 3.** Structural Equation Modeling Diagram From SEP to Survival to Discharge in Patients Who Survived to Admission

Supplement 2.
Data Sharing Statement
