# Supplementary material for: Evaluation of Socioeconomic Position and Survival After Out-of-Hospital Cardiac Arrest in Korea Using Structural Equation Modeling
Source: JAMA Netw Open. 2023 May 10;6(5):e2312722. doi: 10.1001/jamanetworkopen.2023.12722 (PMC10173021; doi:10.1001/jamanetworkopen.2023.12722)
Supplement: Supplement 1. — eMethods. eReferences. eTable 1. Percentage of Missing Data for Each Variable eTable 2. Association Between Individual SEP and Hospital Outcomes in the Total Study Population eTable 3. Mediation Analysis on the Association Between SEP and Survival to Discharge After OHCA (Total Study Population) eTable 4. Estimates of Pathways in the SEP and Survival to Discharge Association (Total Study Population) eTable 5. Characteristics and Outcomes of Patients Who Survived to Admission According to Individual SEP eTable 6. Mediation Analysis on the Association Between SEP and Survival to Discharge After OHCA (Patients Who Survived to Admission) eTable 7. Estimates of Pathways in the SEP and Survival to Discharge Association (Patients Who Survived to Admission) eFigure 1. Study Flowchart eFigure 2. Structural Equation Modeling Diagram From SEP to Survival to Discharge in Total Study Population eFigure 3. Structural Equation Modeling Diagram From SEP to Survival to Discharge in Patients Who Survived to Admission [file jamanetwopen-e2312722-s001.pdf]

Supplemental Online Content

Choi DH, Ro YS, Park JH, et al. Evaluation of socioeconomic position and survival after out-of-hospital cardiac arrest in Korea using structural equation modeling. *JAMA Netw Open.* 2023;6(5):e2312722. doi:10.1001/jamanetworkopen.2023.12722

eMethods.

eReferences.

eTable 1. Percentage of Missing Data for Each Variable

eTable 2. Association Between Individual SEP and Hospital Outcomes in the Total Study Population

eTable 3. Mediation Analysis on the Association Between SEP and Survival to Discharge After OHCA (Total Study Population)

eTable 4. Estimates of Pathways in the SEP and Survival to Discharge Association (Total Study Population)

eTable 5. Characteristics and Outcomes of Patients Who Survived to Admission According to Individual SEP

eTable 6. Mediation Analysis on the Association Between SEP and Survival to Discharge After OHCA (Patients Who Survived to Admission)

eTable 7. Estimates of Pathways in the SEP and Survival to Discharge Association (Patients Who Survived to Admission)

eFigure 1. Study Flowchart

eFigure 2. Structural Equation Modeling Diagram From SEP to Survival to Discharge in Total Study Population

eFigure 3. Structural Equation Modeling Diagram From SEP to Survival to Discharge in Patients Who Survived to Admission

This supplemental material has been provided by the authors to give readers additional information about their work.

© 2023 Choi DH et al. *JAMA Network Open.*

eMethods

Previous evidence supporting the hypothetical model of structural equation modeling analysis

The hypothetical model from socioeconomic position (SEP) to out-of-hospital cardiac arrest (OHCA) survival to discharge was constructed based on prior knowledge and temporal order. Low SEP is associated with living alone and less use of public space, which can influence the possibility of being witnessed at the time of arrest.[1,2] SEP and witnessed status were found to be associated with the provision of bystander cardiopulmonary resuscitation (CPR).[3,4] The initial rhythm of OHCA patients is associated with the mechanism of cardiac arrest and no-flow time (time from arrest to initiation of CPR).[5] Therefore, witnessed status and the provision of bystander CPR, which affects the no-flow time, can affect the initial rhythm. SEP is associated with the accessibility to healthcare facilities, suggesting that SEP may affect the level of receiving emergency department (ED).[6] The SEP of OHCA patients who were successfully resuscitated was found to be associated with the rate of receiving coronary angiography (CAG) and targeted temperature management (TTM).[7,8] Also, hospital factors such as the size and academic status are associated with the use of CAG and TTM.[9,10] Bystander CPR, witnessed status, initial rhythm, ED level, CAG, TTM were found to be associated with the survival of OHCA patients.[11-14]

Age, sex, diabetes mellitus, hypertension, and residential region were included as confounders in the structural equation model. While comorbidities can be explained as either a confounder (a patient with comorbidities may not be able to work the same as a healthy person, thus affecting the SEP) or a mediator (a low SEP patient may have unhealthy lifestyle habits which may increase the occurrence of diseases), we used comorbidities as confounders in accordance with previous literature that observed the association between income and OHCA survival.[3,15]

© 2023 Choi DH et al. *JAMA Network Open*.

eReferences

1. Joo CL, Park JJ, Kim A, Park NL, Lim J, Park HS. Health behaviors and lifestyle patterns of elderly living alone in Korea. *KJFP*. 2019;9(3):247-253. doi:10.21215/kjfp.2019.9.3.247
2. Trawalter S, Hoffman K, Palmer L. Out of place: Socioeconomic status, use of public space, and belonging in higher education. *J Pers Soc Psychol*. 2021;120(1):131-144. doi:10.1037/pspi0000248
3. Moller S, Wissenberg M, Starkopf L, et al. Socioeconomic disparities in prehospital factors and survival after out-of-hospital cardiac arrest. *Heart*. 2021;107(8):627-634. doi:10.1136/heartjnl-2020-317761
4. Munot S, Rugel EJ, Von Huben A, et al. Out-of-hospital cardiac arrests and bystander response by socioeconomic disadvantage in communities of New South Wales, Australia. *Resusc Plus*. 2022;9:100205. doi:10.1016/j.resplu.2022.100205
5. Cournoyer A, Chauny JM, Paquet J, et al. Electrical rhythm degeneration in adults with out-of-hospital cardiac arrest according to the no-flow and bystander low-flow time. *Resuscitation*. 2021;167:355-361. doi:10.1016/j.resuscitation.2021.07.021
6. Cooper RA, Cooper MA, McGinley EL, Fan X, Rosenthal JT. Poverty, wealth, and health care utilization: a geographic assessment. *J Urban Health*. 2012;89(5):828-47. doi:10.1007/s11524-012-9689-3
7. Huebinger R, Abella BS, Chavez S, et al. Socioeconomic status and post-arrest care after out-of-hospital cardiac arrest in Texas. *Resuscitation*. 2022;176:107-116. doi:10.1016/j.resuscitation.2022.03.036
8. Kim TH, Ro YS, Shin SD, et al. Association of health insurance with post-resuscitation care and neurological outcomes after return of spontaneous circulation in out-of-hospital cardiac arrest patients in Korea. *Resuscitation*. 2019;135:176-182. doi:10.1016/j.resuscitation.2018.12.023
9. Bosson N, Tolles J, Shavelle D, et al. Variation in post-cardiac arrest care within a regional EMS system. *Prehosp Emerg Care*. 2022;26(6):772-781. doi:10.1080/10903127.2021.1965681
10. Hanuschak TA, Peng Y, Day A, et al. Patient and hospital factors predict use of coronary angiography in out-of-hospital cardiac arrest patients. *Resuscitation*. 2019;138:182-189. doi:10.1016/j.resuscitation.2019.03.013
11. Bernard SA, Gray TW, Buist MD, et al. Treatment of comatose survivors of out-of-hospital cardiac arrest with induced hypothermia. *N Engl J Med*. 2002;346(8):557-63. doi:10.1056/NEJMoa003289
12. Kempster K, Howell S, Bernard S, et al. Out-of-hospital cardiac arrest outcomes in emergency departments. *Resuscitation*. 2021;166:21-30. doi:10.1016/j.resuscitation.2021.07.003
13. Lahmann AL, Bongiovanni D, Berkefeld A, et al. Predicting factors for long-term survival in patients with out-of-hospital cardiac arrest - A propensity score-matched analysis. *PLoS One*. 2020;15(1):e0218634. doi:10.1371/journal.pone.0218634
14. Wibrandt I, Norsted K, Schmidt H, Schierbeck J. Predictors for outcome among cardiac arrest patients: the importance of initial cardiac arrest rhythm versus time to return of spontaneous circulation, a retrospective cohort study. *BMC Emerg Med*. 2015;15:3. doi:10.1186/s12873-015-0028-3
15. Jonsson M, Harkonen J, Ljungman P, et al. Inequalities in income and education are associated with survival differences after out-of-hospital cardiac arrest: nationwide observational study. *Circulation*. 2021;144(24):1915-1925. doi:10.1161/CIRCULATIONAHA.121.056012

© 2023 Choi DH et al. *JAMA Network Open.*

eTable 1. Percentage of Missing Data for Each Variable

	Total (%)	NHI Q1 (%)	NHI Q2 (%)	NHI Q3 (%)	NHI Q4 (%)	MA (%)
Age	0.0	0.0	0.0	0.0	0.0	0.0
Sex	0.0	0.0	0.0	0.0	0.0	0.0
Diabetes mellitus	0.0	0.0	0.0	0.0	0.0	0.0
Hypertension	0.0	0.0	0.0	0.0	0.0	0.0
Residential region	0.0	0.0	0.0	0.0	0.0	0.0
Location of arrest	2.9	2.8	3.0	2.8	3.2	2.6
Witnessed status	9.0	8.9	8.7	8.7	8.8	11.5
Bystander CPR	2.5	2.5	2.6	2.5	2.4	3.0
Bystander AED use	1.4	1.3	1.4	1.3	1.3	1.6
RTI	0.2	0.2	0.2	0.3	0.2	0.2
Initial rhythm	0.6	0.6	0.6	0.6	0.6	0.5
ED level	0.0	0.0	0.0	0.0	0.0	0.0
CAG	0.0	0.0	0.0	0.0	0.0	0.0
TTM	0.0	0.0	0.0	0.0	0.0	0.0
Survival to admission	0.0	0.0	0.0	0.0	0.0	0.0
Survival to discharge	0.0	0.0	0.0	0.0	0.0	0.0
Good neurological recovery	0.0	0.0	0.0	0.0	0.0	0.0

Abbreviations: NHI, National Health Insurance; MA, medical aid; CPR, cardiopulmonary resuscitation; AED, automated external defibrillator; RTI, response time interval; ED, emergency department; CAG, coronary angiography; TTM, targeted temperature management.

© 2023 Choi DH et al. *JAMA Network Open*.

eTable 2. Association Between Individual SEP and Hospital Outcomes in the Total Study Population

	Survival to discharge		Good neurological recovery	
	Crude OR (95% CI)	Adjusted OR (95% CI)	Crude OR (95% CI)	Adjusted OR (95% CI)
**MA vs. NHI Q1–4**				
NHI Q1–4	Reference	Reference	Reference	Reference
MA	0.55 (0.50–0.60)	0.56 (0.53–0.59)	0.40 (0.35–0.46)	0.41 (0.38–0.45)
**MA + NHI Q4 vs. NHI Q1–3**				
NHI Q1–3	Reference	Reference	Reference	Reference
MA + NHI Q4	0.89 (0.84–0.93)	0.83 (0.79–0.87)	0.79 (0.74–0.84)	0.72 (0.68–0.77)
**MA + NHI Q3–4 vs. NHI Q1–2**				
NHI Q1–2	Reference	Reference	Reference	Reference
MA + NHI Q3–4	1.01 (0.97–1.05)	0.84 (0.80–0.88)	0.97 (0.92–1.02)	0.76 (0.72–0.81)
**MA + NHI Q2–4 vs. NHI Q1**				
NHI Q1	Reference	Reference	Reference	Reference
MA + NHI Q2–4	1.11 (1.06–1.16)	0.87 (0.83–0.92)	1.10 (1.04–1.16)	0.80 (0.75–0.85)

Adjusted odds ratios were calculated with a multivariable logistic regression model adjusting for age, sex, hypertension, diabetes mellitus, and residential region.
Abbreviations: SEP, socioeconomic position; OR, odds ratio; CI, confidence interval; NHI, National Health Insurance; MA, medical aid.

© 2023 Choi DH et al. *JAMA Network Open.*

eTable 3. Mediation Analysis on the Association Between SEP and Survival to Discharge After OHCA (Total Study Population)

SEP	Possible mediator	Adjusted OR (95% CI)			Mediation analysis result
		SEP → Mediator[a]	Mediator → Survival to discharge[a]	SEP → Survival to discharge[b]	
**MA vs. NHI Q1–4**					
	Witnessed arrest (vs. unwitnessed arrest)	0.81 (0.77–0.84)	5.15 (4.87–5.46)	0.61 (0.56–0.68)	Mediator
	Bystander CPR (vs. no bystander CPR)	0.82 (0.79–0.86)	1.97 (1.87–2.07)	0.58 (0.53–0.64)	Mediator
	Bystander AED (vs. no bystander AED)	1.01 (0.89–1.15)[c]	1.39 (1.24–1.57)	0.56 (0.51–0.62)	Not a mediator
	RTI < 8 minutes (vs. ≥ 8 minutes)	1.04 (1.00–1.08)[c]	1.60 (1.53–1.67)	0.56 (0.50–0.61)	Not a mediator
	Initial shockable rhythm (vs. initial non-shockable rhythm)	0.59 (0.55–0.63)	10.76 (10.25–11.28)	0.72 (0.65–0.80)	Mediator
	ED level 1–2 (vs. ED level 3–4)	0.77 (0.74–0.81)	3.52 (3.31–3.74)	0.59 (0.54–0.65)	Mediator
**MA + NHI Q4 vs. NHI Q1–3**					
	Witnessed arrest (vs. unwitnessed arrest)	0.90 (0.88–0.93)	5.15 (4.87–5.46)	0.87 (0.83–0.92)	Mediator
	Bystander CPR (vs. no bystander CPR)	0.89 (0.87–0.92)	1.97 (1.87–2.07)	0.85 (0.81–0.89)	Mediator
	Bystander AED (vs. no bystander AED)	0.94 (0.86–1.02)[c]	1.39 (1.24–1.57)	0.83 (0.79–0.87)	Not a mediator
	RTI < 8 minutes (vs. ≥ 8 minutes)	1.04 (1.01–1.07)	1.60 (1.53–1.67)	0.83 (0.79–0.87)	Mediator
	Initial shockable rhythm (vs. initial non-shockable rhythm)	0.87 (0.84–0.91)	10.76 (10.25–11.28)	0.90 (0.85–0.95)	Mediator
	ED level 1–2 (vs. ED level 3–4)	0.92 (0.89–0.94)	3.52 (3.31–3.74)	0.85 (0.81–0.89)	Mediator

© 2023 Choi DH et al. *JAMA Network Open.*

**MA + NHI Q3–4 vs. NHI Q1–2**				
Witnessed arrest (vs. unwitnessed arrest)	0.90 (0.88–0.92)	5.15 (4.87–5.46)	0.88 (0.84–0.92)	Mediator
Bystander CPR (vs. no bystander CPR)	0.89 (0.87–0.92)	1.97 (1.87–2.07)	0.86 (0.82–0.90)	Mediator
Bystander AED (vs. no bystander AED)	0.94 (0.87–1.01)[c]	1.39 (1.24–1.57)	0.84 (0.80–0.88)	Not a mediator
RTI < 8 minutes (vs. ≥ 8 minutes)	1.01 (0.99–1.04)[c]	1.60 (1.53–1.67)	0.84 (0.80–0.87)	Not a mediator
Initial shockable rhythm (vs. initial non-shockable rhythm)	0.90 (0.88–0.93)	10.76 (10.25–11.28)	0.89 (0.85–0.93)	Mediator
ED level 1–2 (vs. ED level 3–4)	0.91 (0.89–0.93)	3.52 (3.31–3.74)	0.86 (0.82–0.90)	Mediator
**MA + NHI Q2–4 vs. NHI Q1**				
Witnessed arrest (vs. unwitnessed arrest)	0.91 (0.89–0.93)	5.15 (4.87–5.46)	0.92 (0.87–0.96)	Mediator
Bystander CPR (vs. no bystander CPR)	0.90 (0.88–0.92)	1.97 (1.87–2.07)	0.90 (0.85–0.94)	Mediator
Bystander AED (vs. no bystander AED)	0.92 (0.85–0.99)	1.39 (1.24–1.57)	0.87 (0.83–0.92)	Mediator
RTI < 8 minutes (vs. ≥ 8 minutes)	0.99 (0.97–1.02)[c]	1.60 (1.53–1.67)	0.87 (0.83–0.91)	Not a mediator
Initial shockable rhythm (vs. initial non-shockable rhythm)	0.94 (0.91–0.97)	10.76 (10.25–11.28)	0.90 (0.86–0.95)	Mediator
ED level 1–2 (vs. ED level 3–4)	0.88 (0.86–0.90)	3.52 (3.31–3.74)	0.90 (0.86–0.95)	Mediator

[a]Adjusted odds ratios were calculated with a multivariable logistic regression model adjusting for age, sex, hypertension, diabetes mellitus, and residential region.
[b]Adjusted odds ratios were calculated with a multivariable logistic regression model adjusting for the possible mediator along with age, sex, hypertension, diabetes mellitus, and residential region.
[c]Nonsignificant (p-value>0.05) results.
Abbreviations: SEP, socioeconomic position; OHCA, out-of-hospital cardiac arrest; OR, odds ratio; CI, confidence interval; MA, medical aid; NHI, National Health Insurance; CPR, cardiopulmonary resuscitation; AED, automated external defibrillator; RTI, response time interval; ED, emergency department.

© 2023 Choi DH et al. *JAMA Network Open.*

eTable 4. Estimates of Pathways in the SEP and Survival to Discharge Association (Total Study Population)

Pathway	Effect		Mediation proportion (95% CI)
	Estimate	95% CI	
**MA + NHI Q4 vs. NHI Q1–3**			
Total	-0.013	-0.016, -0.009	-
Witnessed status	-0.002	-0.003, -0.002	18.0 (12.1–23.9)
Bystander CPR	-0.001	-0.001, -0.001	7.0 (4.6–9.4)
Bystander CPR but not through witnessed status[a]	-0.001	-0.001, -0.001	6.6 (4.2–8.9)
Initial rhythm	-0.004	-0.005, -0.003	31.6 (21.9–41.3)
Initial rhythm but not through witnessed status or bystander CPR[a]	-0.003	-0.004, -0.002	22.0 (13.4–30.6)
ED level	-0.001	-0.001, -0.001	7.9 (4.9–10.9)
Direct	-0.006	-0.009, -0.003	45.5 (31.4–59.6)
**MA + NHI Q3–4 vs. NHI Q1–2**			
Total	-0.012	-0.015, -0.009	-
Witnessed status	-0.002	-0.003, -0.002	18.9 (13.2–24.6)
Bystander CPR	-0.001	-0.001, -0.001	7.0 (4.7–9.3)
Bystander CPR but not through witnessed status[a]	-0.001	-0.001, -0.001	6.6 (4.4–8.8)
Initial rhythm	-0.003	-0.004, -0.002	25.3 (16.9–33.6)
Initial rhythm but not through witnessed status or bystander CPR[a]	-0.002	-0.003, -0.001	15.3 (7.7–23.0)
ED level	-0.001	-0.001, -0.001	9.1 (6.1–12.2)
Direct	-0.006	-0.009, -0.003	50.1 (37.6–62.6)
**MA + NHI Q2–4 vs. NHI Q1**			
Total	-0.009	-0.012, -0.006	
Witnessed status	-0.002	-0.003, -0.002	23.5 (14.1–32.9)
Bystander CPR	-0.001	-0.001, -0.001	9.0 (5.2–12.8)
Bystander CPR but not through witnessed status[a]	-0.001	-0.001, -0.001	8.5 (4.8–12.1)
Initial rhythm	-0.001	-0.002, 0.000	-[b]
Initial rhythm but not through witnessed status or bystander CPR[a]	0.000	-0.001, 0.001	-[b]
ED level	-0.001	-0.002, -0.001	16.6 (10.2–23.0)
Direct	-0.005	-0.008, -0.002	56.0 (39.6–72.3)

© 2023 Choi DH et al. *JAMA Network Open.*

[a]The proportion mediated through a mediator but not through its intermediate confounders (variable that is affected by the exposure, which in turn affects the outcome and mediator) is assessed.
[b]The mediation proportions are not calculated for nonsignificant estimates.
Abbreviations: SEP, socioeconomic position; CI, confidence interval; MA, medical aid; NHI, National Health Insurance; CPR, cardiopulmonary resuscitation; ED, emergency department.

© 2023 Choi DH et al. *JAMA Network Open.*

eTable 5. Characteristics and Outcomes of Patients Who Survived to Admission According to Individual SEP

	NHI Q1	NHI Q2	NHI Q3	NHI Q4	MA	p-value
Total	7,680	5,546	4,683	4,711	1,625	
Age, years	69 (55–78)	63 (52–73)	59 (50–70)	62 (53–73)	65 (54–77)	<0.001
Standardized mean difference	Reference	-0.29	-0.45	-0.27	-0.09	
Age, 65–120 years old	4,403 (57.3)	2,525 (45.5)	1,719 (36.7)	2,097 (44.5)	821 (50.5)	<0.001
Sex						<0.001
Male	5,146 (67.0)	3,947 (71.2)	3,365 (71.9)	3,278 (69.6)	921 (56.7)	
Female	2,534 (33.0)	1,599 (28.8)	1,318 (28.1)	1,433 (30.4)	704 (43.3)	
Diabetes mellitus	1,870 (24.3)	1,228 (22.1)	966 (20.6)	1,112 (23.6)	468 (28.8)	<0.001
Hypertension	3,753 (48.9)	2,445 (44.1)	1,940 (41.4)	2,132 (45.3)	791 (48.7)	<0.001
Residential region, metropolitan	3,806 (49.6)	2,744 (49.5)	2,368 (50.6)	2,280 (48.4)	789 (48.6)	0.29
Location of arrest, public	1,921 (25.0)	1,496 (27.0)	1,362 (29.1)	1,369 (29.1)	289 (17.8)	<0.001
Witnessed arrest	5,891 (76.7)	4,217 (76.0)	3,466 (74.0)	3,539 (75.1)	1,121 (69.0)	<0.001
Bystander CPR	5,545 (72.2)	3,964 (71.5)	3,311 (70.7)	3,316 (70.4)	1,095 (67.4)	<0.001
Bystander AED use	249 (3.2)	188 (3.4)	141 (3.0)	133 (2.8)	55 (3.4)	0.48
RTI, minutes	6 (5–9)	6 (5–9)	6 (5–9)	6 (5–8)	6 (5–8)	0.22
Standardized mean difference	Reference	0.01	0	-0.01	-0.04	
RTI < 8 minutes	5,004 (65.2)	3,579 (64.5)	2,963 (63.3)	3,112 (66.1)	1,088 (67.0)	0.02
Initial shockable rhythm	2,717 (35.4)	2,161 (39.0)	1,878 (40.1)	1,743 (37.0)	366 (22.5)	<0.001
ED level 1–2	5,961 (77.6)	4,255 (76.7)	3,610 (77.1)	3,651 (77.5)	1,142 (70.3)	<0.001
CAG	1,717 (22.4)	1,309 (23.6)	1,128 (24.1)	1,086 (23.1)	207 (12.7)	<0.001
TTM	1,098 (14.3)	772 (13.9)	687 (14.7)	641 (13.6)	150 (9.2)	<0.001
Survival to discharge	2,916 (38.0)	2,250 (40.6)	1,880 (40.1)	1,832 (38.9)	465 (28.6)	<0.001
Good neurological recovery	1,842 (24.0)	1,458 (26.3)	1,250 (26.7)	1,108 (23.5)	216 (13.3)	<0.001

© 2023 Choi DH et al. *JAMA Network Open.*

Categorical variables are presented as numbers (proportions) and continuous variables are presented as median (interquartile ranges).
Abbreviations: SEP, socioeconomic position; NHI, National Health Insurance; MA, medical aid; CPR, cardiopulmonary resuscitation; AED, automated external defibrillator; RTI, response time interval; ED, emergency department; CAG, coronary angiography; TTM, targeted temperature management.

© 2023 Choi DH et al. *JAMA Network Open.*

eTable 6. Mediation Analysis on the Association Between SEP and Survival to Discharge After OHCA (Patients Who Survived to Admission)

SEP	Possible mediator	Adjusted OR (95% CI)			Mediation analysis result
		SEP → Mediator[a]	Mediator → Survival to discharge[a]	SEP → Survival to discharge[b]	
**MA vs. NHI Q1–4**					
	Witnessed arrest (vs. unwitnessed arrest)	0.72 (0.65–0.81)	2.26 (2.11–2.42)	0.72 (0.64–0.80)	Mediator
	Bystander CPR (vs. no bystander CPR)	0.82 (0.74–0.92)	1.57 (1.47–1.66)	0.69 (0.62–0.78)	Mediator
	Bystander AED (vs. no bystander AED)	1.10 (0.83–1.45)[c]	1.18 (1.02–1.37)	0.68 (0.61–0.76)	Not a mediator
	RTI < 8 minutes (vs. ≥ 8 minutes)	1.11 (1.00–1.24)[c]	1.27 (1.20–1.34)	0.68 (0.61–0.76)	Not a mediator
	Initial shockable rhythm (vs. initial non-shockable rhythm)	0.52 (0.46–0.59)	6.43 (6.05–6.83)	0.84 (0.74–0.95)	Mediator
	ED level 1–2 (vs. ED level 3–4)	0.72 (0.64–0.80)	2.46 (2.30–2.64)	0.72 (0.64–0.80)	Mediator
	CAG (vs. no CAG)	0.53 (0.45–0.61)	5.41 (5.06–5.80)	0.78 (0.69–0.88)	Mediator
	TTM (vs. no TTM)	0.64 (0.54–0.77)	2.07 (1.92–2.23)	0.70 (0.63–0.79)	Mediator
**MA + NHI Q4 vs. NHI Q1–3**					
	Witnessed arrest (vs. unwitnessed arrest)	0.89 (0.83–0.95)	2.26 (2.11–2.42)	0.90 (0.84–0.96)	Mediator
	Bystander CPR (vs. no bystander CPR)	0.90 (0.85–0.96)	1.57 (1.47–1.66)	0.89 (0.84–0.95)	Mediator
	Bystander AED (vs. no bystander AED)	0.92 (0.78–1.09)[c]	1.18 (1.02–1.37)	0.88 (0.83–0.94)	Not a mediator
	RTI < 8 minutes (vs. ≥ 8 minutes)	1.09 (1.03–1.16)	1.27 (1.20–1.34)	0.88 (0.83–0.94)	Mediator

© 2023 Choi DH et al. *JAMA Network Open.*

Initial shockable rhythm (vs. initial non-shockable rhythm)	0.82 (0.77–0.87)	6.43 (6.05–6.83)	0.95 (0.88–1.01)	Mediator
ED level 1–2 (vs. ED level 3–4)	0.92 (0.86–0.98)	2.46 (2.30–2.64)	0.89 (0.84–0.95)	Mediator
CAG (vs. no CAG)	0.86 (0.80–0.92)	5.41 (5.06–5.80)	0.91 (0.86–0.97)	Mediator
TTM (vs. no TTM)	0.86 (0.79–0.94)	2.07 (1.92–2.23)	0.89 (0.84–0.95)	Mediator
**MA + NHI Q3–4 vs. NHI Q1–2**				
Witnessed arrest (vs. unwitnessed arrest)	0.85 (0.80–0.90)	2.26 (2.11–2.42)	0.90 (0.85–0.95)	Mediator
Bystander CPR (vs. no bystander CPR)	0.89 (0.84–0.94)	1.57 (1.47–1.66)	0.89 (0.84–0.94)	Mediator
Bystander AED (vs. no bystander AED)	0.89 (0.77–1.03)[c]	1.18 (1.02–1.37)	0.88 (0.83–0.93)	Not a mediator
RTI < 8 minutes (vs. ≥ 8 minutes)	1.01 (0.96–1.06)[c]	1.27 (1.20–1.34)	0.88 (0.83–0.93)	Not a mediator
Initial shockable rhythm (vs. initial non-shockable rhythm)	0.88 (0.84–0.93)	6.43 (6.05–6.83)	0.91 (0.86–0.97)	Mediator
ED level 1–2 (vs. ED level 3–4)	0.92 (0.86–0.97)	2.46 (2.30–2.64)	0.89 (0.84–0.94)	Mediator
CAG (vs. no CAG)	0.89 (0.84–0.95)	5.41 (5.06–5.80)	0.89 (0.84–0.95)	Mediator
TTM (vs. no TTM)	0.91 (0.84–0.98)	2.07 (1.92–2.23)	0.88 (0.84–0.93)	Mediator
**MA + NHI Q2–4 vs. NHI Q1**				
Witnessed arrest (vs. unwitnessed arrest)	0.87 (0.81–0.92)	2.26 (2.11–2.42)	0.92 (0.87–0.98)	Mediator
Bystander CPR (vs. no bystander CPR)	0.89 (0.84–0.94)	1.57 (1.47–1.66)	0.92 (0.86–0.97)	Mediator
Bystander AED (vs. no bystander AED)	0.95 (0.81–1.11)[c]	1.18 (1.02–1.37)	0.90 (0.85–0.96)	Not a mediator

© 2023 Choi DH et al. *JAMA Network Open.*

RTI < 8 minutes (vs. ≥ 8 minutes)	0.99 (0.94–1.05)[c]	1.27 (1.20–1.34)	0.90 (0.85–0.96)	Not a mediator
Initial shockable rhythm (vs. initial non-shockable rhythm)	0.94 (0.88–1.00)	6.43 (6.05–6.83)	0.93 (0.87–0.99)	Mediator
ED level 1–2 (vs. ED level 3–4)	0.89 (0.83–0.95)	2.46 (2.30–2.64)	0.92 (0.87–0.98)	Mediator
CAG (vs. no CAG)	0.91 (0.85–0.98)	5.41 (5.06–5.80)	0.92 (0.86–0.98)	Mediator
TTM (vs. no TTM)	0.89 (0.82–0.97)	2.07 (1.92–2.23)	0.91 (0.86–0.97)	Mediator

[a]Adjusted odds ratios were calculated with a multivariable logistic regression model adjusting for age, sex, hypertension, diabetes mellitus, and residential region.
[b]Adjusted odds ratios were calculated with a multivariable logistic regression model adjusting for the possible mediator along with age, sex, hypertension, diabetes mellitus, and residential region.
[c]Nonsignificant (p-value>0.05) results.
Abbreviations: SEP, socioeconomic position; OHCA, out-of-hospital cardiac arrest; OR, odds ratio; CI, confidence interval; MA, medical aid; NHI, National Health Insurance; CPR, cardiopulmonary resuscitation; AED, automated external defibrillator; RTI, response time interval; ED, emergency department; CAG, coronary angiography; TTM, targeted temperature management.

© 2023 Choi DH et al. *JAMA Network Open.*

eTable 7. Estimates of Pathways in the SEP and Survival to Discharge Association (Patients Who Survived to Admission)

Pathway	Effect		Mediation proportion (95% CI)
	Estimate	95% CI	
**MA + NHI Q4 vs. NHI Q1–3**			
Total	-0.030	-0.043, -0.016	-
Witnessed status	-0.003	-0.005, -0.002	11.7 (3.9–19.5)
Bystander CPR	-0.002	-0.003, -0.001	5.5 (1.4–9.6)
Bystander CPR but not through witnessed status[a]	-0.001	-0.002, 0.000	4.9 (1.0–8.9)
Initial rhythm	-0.017	-0.022, -0.011	55.5 (31.1–80.0)
Initial rhythm but not through witnessed status or bystander CPR[a]	-0.014	-0.020, -0.009	48.7 (26.2–71.2)
ED level	-0.002	-0.004, 0.000	7.3 (1.0–13.5)
CAG	-0.005	-0.008, -0.003	17.4 (8.0–26.8)
CAG but not through initial rhythm or ED level[a]	-0.002	-0.004, 0.000	-[b]
TTM	-0.001	-0.002, -0.001	4.8 (1.5–8.1)
TTM but not through ED level[a]	-0.001	-0.002, 0.000	4.1 (1.0–7.2)
Direct	-0.005	-0.017, 0.007	-[b]
**MA + NHI Q3–4 vs. NHI Q1–2**			
Total	-0.028	-0.040, -0.016	-
Witnessed status	-0.005	-0.006, -0.003	16.2 (7.7–24.6)
Bystander CPR	-0.002	-0.003, -0.001	6.5 (2.4–10.5)
Bystander CPR but not through witnessed status[a]	-0.002	-0.002, -0.001	5.6 (1.8–9.5)
Initial rhythm	-0.010	-0.015, -0.006	36.9 (19.4–54.3)
Initial rhythm but not through witnessed status or bystander CPR[a]	-0.008	-0.012, -0.003	28.0 (12.1–43.8)
ED level	-0.002	-0.004, -0.001	7.6 (1.8–13.4)
CAG	-0.004	-0.006, -0.002	13.6 (5.7–21.4)
CAG but not through initial rhythm or ED level[a]	-0.001	-0.003, 0.000	-[b]
TTM	-0.001	-0.002, 0.000	3.2 (0.5–6.0)
TTM but not through ED level[a]	-0.001	-0.001, 0.000	-[b]

© 2023 Choi DH et al. *JAMA Network Open.*

Direct	-0.010	-0.021, 0.001	34.9 (9.3–60.5)
**MA + NHI Q2–4 vs. NHI Q1**			
Total	-0.018	-0.031, -0.005	-
Witnessed status	-0.004	-0.006, -0.002	22.7 (4.9–40.5)
Bystander CPR	-0.002	-0.003, -0.001	10.3 (1.5–19.0)
Bystander CPR but not through witnessed status[a]	-0.002	-0.003, -0.001	9.1 (1.0–17.2)
Initial rhythm	-0.005	-0.010, -0.001	30.6 (3.4–57.8)
Initial rhythm but not through witnessed status or bystander CPR[a]	-0.003	-0.008, 0.002	-[b]
ED level	-0.003	-0.005, -0.001	16.1 (2.6–29.5)
CAG	-0.003	-0.005, -0.001	17.2 (2.8–31.6)
CAG but not through initial rhythm or ED level[a]	-0.001	-0.003, 0.001	-[b]
TTM	-0.001	-0.002, 0.000	6.1 (0.3–12.0)
TTM but not through ED level[a]	-0.001	-0.002, 0.000	-[b]
Direct	-0.004	-0.015, 0.007	-[b]

[a]The proportion mediated through a mediator but not through its intermediate confounders (variable that is affected by the exposure, which in turn affects the outcome and mediator) is assessed.
[b]The mediation proportions are not calculated for nonsignificant estimates.
Abbreviations: SEP, socioeconomic position; MA, medical aid; NHI, National Health Insurance; CI, confidence interval; CPR, cardiopulmonary resuscitation; ED, emergency department; CAG, coronary angiography; TTM, targeted temperature management.

© 2023 Choi DH et al. *JAMA Network Open.*

eFigure 1. Study Flowchart

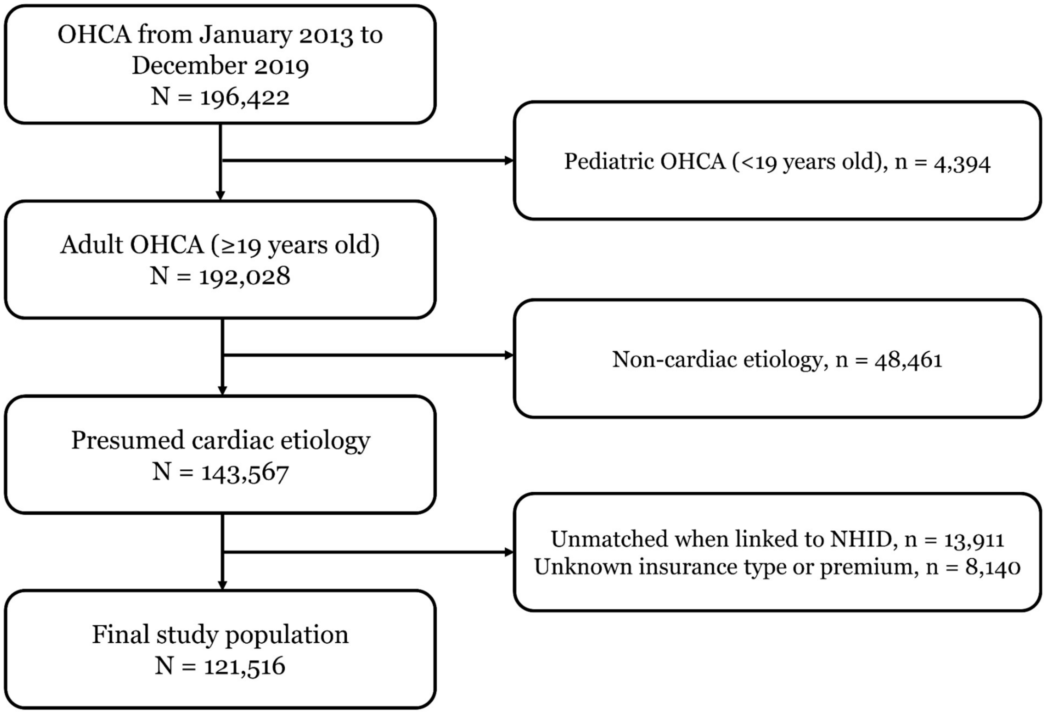

Abbreviations: OHCA, out-of-hospital cardiac arrest; NHID, National Health Insurance Database.

© 2023 Choi DH et al. *JAMA Network Open*.

eFigure 2. Structural Equation Modeling Diagram From SEP to Survival to Discharge in Total Study Population

SEP was binarized into (A) MA + NHI Q4 vs. NHI Q1–3, (B) MA + NHI Q3–4 vs. NHI Q1–2, and (C) MA + NHI Q2–4 vs. NHI Q1. Coefficients (standard errors) of pathways between SEP, survival to discharge, and mediators are shown in the figure. All models were well fitted ((A) RMSEA=0.057, SRMR=0.014, GFI=1.00, and CFI=0.98; (B) RMSEA=0.057, SRMR=0.014, GFI=1.00, and CFI=0.98; (C) RMSEA=0.057, SRMR=0.014, GFI=1.00, and CFI=0.98). Abbreviations: SEP, socioeconomic position; MA, medical aid; NHI, National Health Insurance; CPR, cardiopulmonary resuscitation; ED, emergency department.

© 2023 Choi DH et al. *JAMA Network Open*.

(A)

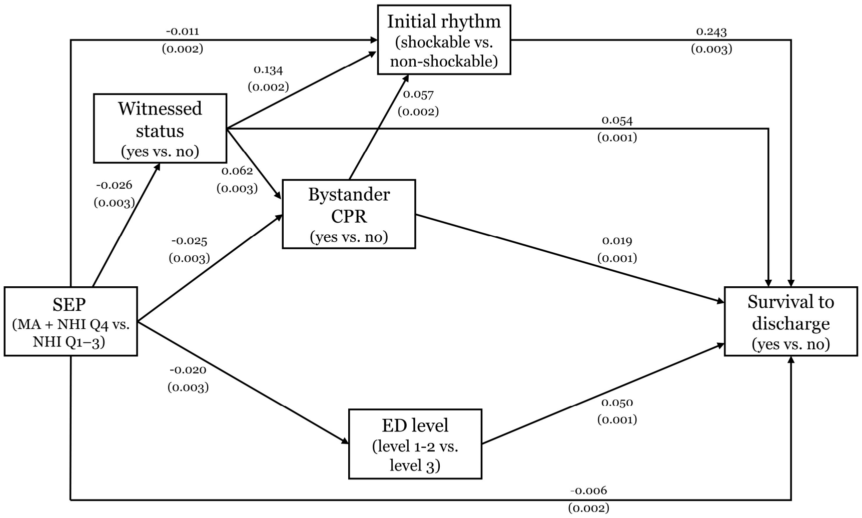

(B)

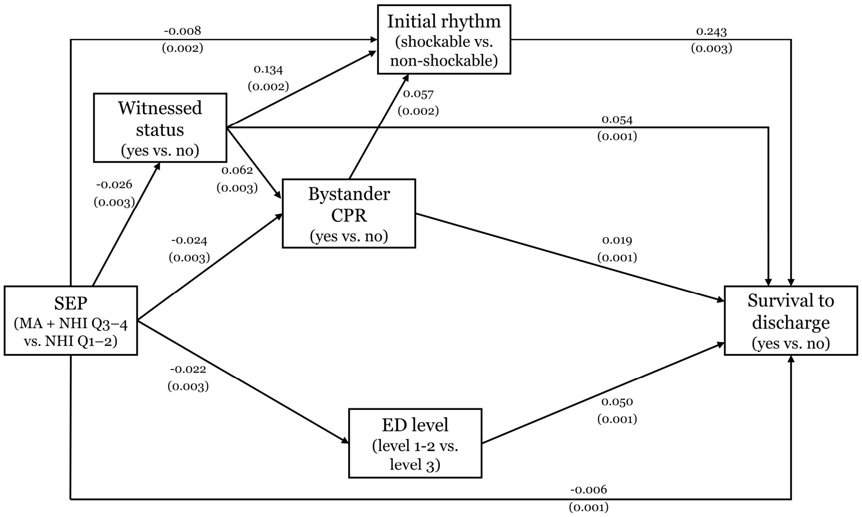

(C)

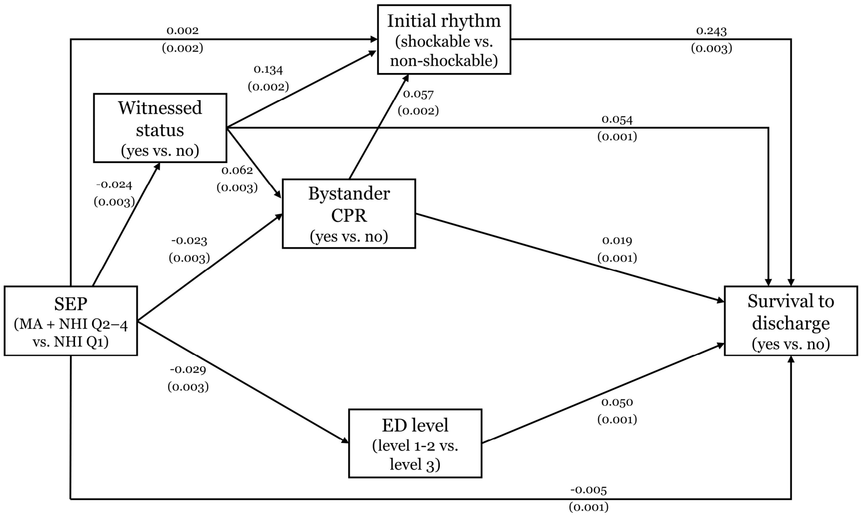

© 2023 Choi DH et al. *JAMA Network Open*.

eFigure 3. Structural Equation Modeling Diagram From SEP to Survival to Discharge in Patients Who Survived to Admission

SEP was binarized into (A) MA + NHI Q4 vs. NHI Q1–3, (B) MA + NHI Q3–4 vs. NHI Q1–2, and (C) MA + NHI Q2–4 vs. NHI Q1. Coefficients (standard errors) of pathways between SEP, survival to discharge, and mediators are shown in the figure. All models were well fitted ((A) RMSEA=0.048, SRMR=0.019, GFI=1.00, and CFI=0.98; (B) RMSEA=0.048, SRMR=0.019, GFI=1.00, and CFI=0.98; (C) RMSEA=0.048, SRMR=0.019, GFI=1.00, and CFI=0.98). Abbreviations: SEP, socioeconomic position; MA, medical aid; NHI, National Health Insurance; CPR, cardiopulmonary resuscitation; ED, emergency department; CAG, coronary angiography; TTM, targeted temperature management.

© 2023 Choi DH et al. *JAMA Network Open*.

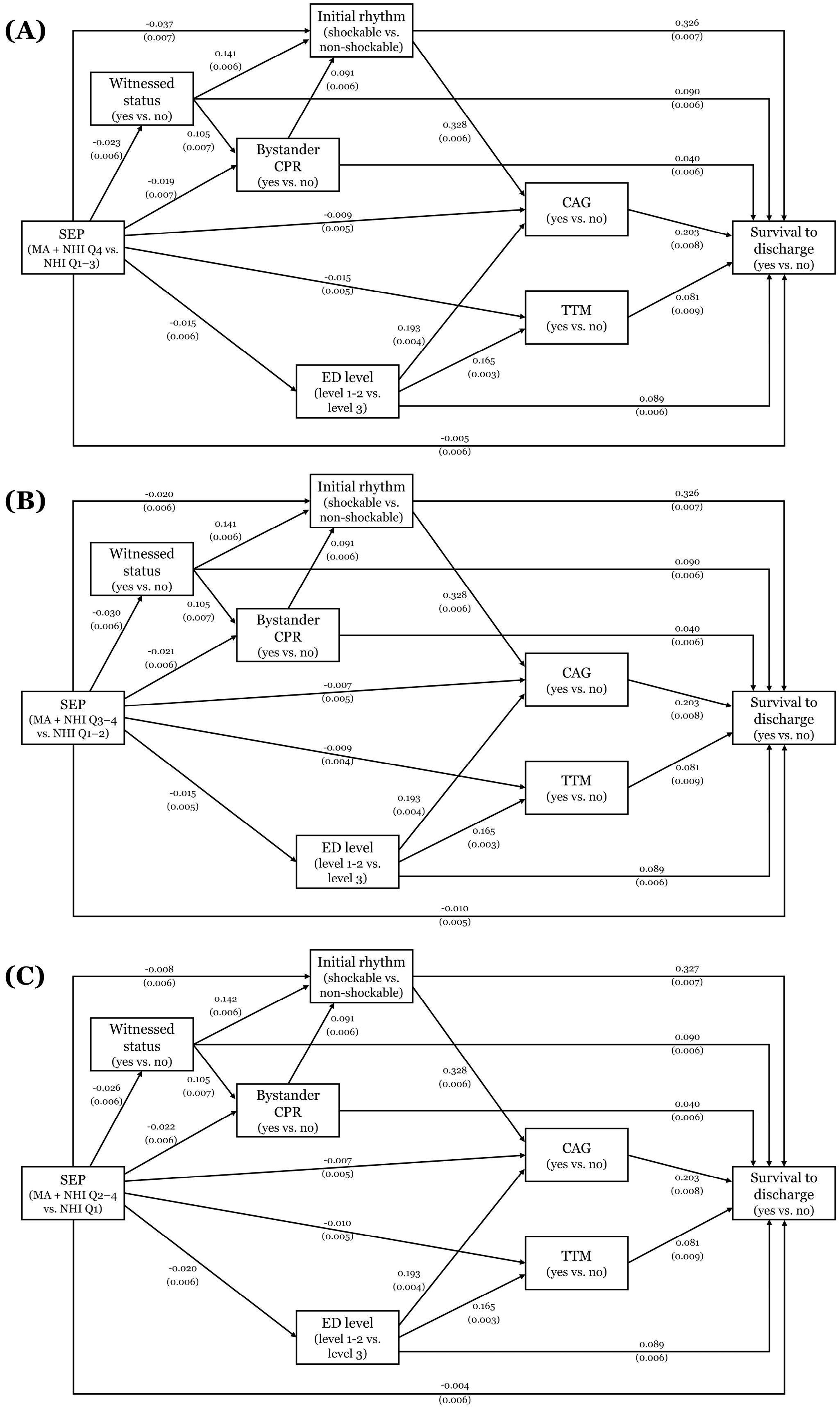

© 2023 Choi DH et al. *JAMA Network Open*.
