# Supplementary material for: Evaluation of Socioeconomic Position and Survival After Out-of-Hospital Cardiac Arrest in Korea Using Structural Equation Modeling
Source: JAMA Netw Open. 2023 May 10;6(5):e2312722. doi: 10.1001/jamanetworkopen.2023.12722 (PMC10173021; doi:10.1001/jamanetworkopen.2023.12722)
Supplement: Supplement 2. — Data Sharing Statement [file jamanetwopen-e2312722-s002.pdf]

Data Sharing Statement

Choi. Evaluation of Socioeconomic Position and Survival After Out-of-Hospital Cardiac Arrest in Korea Using Structural Equation Modeling. *JAMA Netw Open*. Published May 10, 2023. doi:10.1001/jamanetworkopen.2023.12722

Data
Data available: No

Additional Information
Explanation for why data not available: The data for this study was obtained from the Korean National Health Insurance Service and Korean Centers for Disease Control and Prevention. Restrictions apply to the availability of these data and so are not publicly available, but may be available from the corresponding author on reasonable request.
